# Effect of vaccination age on cost-effectiveness of human papillomavirus vaccination against cervical cancer in China

**DOI:** 10.1186/s12885-016-2207-3

**Published:** 2016-02-26

**Authors:** Yi-Jun Liu, Qian Zhang, Shang-Ying Hu, Fang-Hui Zhao

**Affiliations:** Department of Cancer Epidemiology, Cancer Hospital, Chinese Academy of Medical Sciences (CAMS) & Peking Union Medical College (PUMC), 17 South Panjiayuan Lane, P.O. Box 2258, Beijing, 100021 China; Department of Preventive Medicine, School of Public Health, Zunyi Medical College, 201 Dalian Road, Zunyi, 563099 China

**Keywords:** Cervical cancer, HPV vaccine, Cost-effectiveness, Vaccine age, Catch-up

## Abstract

**Background:**

The cost-effectiveness of human papillomavirus (HPV) vaccination in women pre-sexual debut has been demonstrated in many countries. This study aimed to estimate the cost-effectiveness of a 3-dose bivalent HPV vaccination at ages 12 to 55 year in both rural and urban settings in China.

**Methods:**

The Markov cohort model simulated the natural history of HPV infection and included the effect of screening and HPV vaccination over the lifetime of a 100,000 female cohort. Transition probabilities and utilities were obtained from published literature. Cost data were estimated by Delphi panel using healthcare payers’ perspective. Vaccine cost was assumed Hong Kong listed price. Vaccine efficacy (VE) was based on the PATRICIA trial data assuming VE irrespective of HPV type at all ages on incident HPV. Costs and outcomes were discounted at 3 %. Cervical cancer cases and incremental cost-effectiveness ratio (ICER) for vaccination and screening compared with screening alone were estimated for each vaccination age. Reduced VE in women post-sexual debut were investigated in scenario analyses.

**Results:**

With 70 % vaccination coverage, a reduction of cancer cases varying from 585 to 33 in rural and 691 to 32 in urban were estimated at ages 12 to 55, respectively. The discounted ICERs of vaccination at any age under 23 years in rural and any age under 25 years in urban were lower than the current threshold. Scenario analyses with lower VE post-sexual debut confirmed the results with age 20 in rural and 21 in urban had consistent lower ICERs. The more ‘catch-up’ cohorts vaccinated at the start of a program, the more cancer lesions are avoided in the long-term.

**Conclusions:**

Vaccination at any age under 23 years old in rural and any age under 25 years old in urban were cost-effective. Catch-up to the age of 25 years in rural and urban could still be cost-effective.

**Electronic supplementary material:**

The online version of this article (doi:10.1186/s12885-016-2207-3) contains supplementary material, which is available to authorized users.

## Background

Cervical cancer (CC) is an important cause of morbidity and mortality among Chinese women. It is estimated the age-standardized incidence and mortality rates were 7.5 and 3.4 per 100,000 women in China in 2012, lower than corresponding world statistics, 14.0 and 6.8 per 100,000 [1]. However, given the large population base, China accounted for 11.7 % (62,000 new cases) of the world’s annual CC cases and 11.3 % (30,000 deaths) of the world’s annual CC deaths [1]. Although organized screening programs can reduce the cervical cancer burden through early detection and treatment of pre-cancerous lesions, the effectiveness of cervical cancer screening in China is compromised due to dysfunctional health care infrastructure, national screening program being only been made accessible to a limited population in rural China, and wide disparities in access to health care in rural areas.

A new opportunity to reduce preventable deaths from cervical cancer is the use of HPV vaccination. Two prophylactic HPV vaccines are available since 2007 and have high efficacy (>90 %) for preventing high-grade cervical lesions associated with HPV-16 and HPV-18 [2–3]. So far, there are more than 160 countries which have approved the prophylactic vaccines and many have gradually introduced the vaccines into the national routine immunization program [4]. To date, phase III clinical trials on a 3 doses of bivalent HPV vaccination (3DBV) have been ongoing for over 7 years in mainland China [5]. Though at this stage, it is uncertain whether a full-scale initiative to vaccinate girls in China will be available [6], we expect that it will be approved by the State Food and Drug Administration in the foreseeable future.

The constant growth of healthcare demand, in an economic context characterized by limited resources, requires that the decision-making process is based on the comparison of alternative choices [7]. The World Health Organization (WHO) recommends that the cost-effectiveness of introducing a new vaccine to the national immunization program is considered before such a strategy is implemented [8], and has reiterated this advice for the case of HPV vaccination [9]. To date, epidemiological and economic models to determine the cost-effectiveness of HPV vaccines have been used by government policy-makers in many countries. Cost-effectiveness analysis based on modelling studies can integrate currently available clinical data with country-specific epidemiological data to evaluate the potential long-term impact of adding vaccination to screening [10]. To inform policy-making around HPV 16/18 vaccination, multiple studies have been done in different countries exploring the health and economic significance of HPV vaccination [11–15], and consistently shown introduction of an HPV vaccine could be cost-effective compared with current practice, even though incremental cost-effectiveness varies widely with varying degrees of complexity and transparency of each model [16].

Our previous research have already estimated the incremental clinical and economic impact of HPV vaccination in addition to screening compared with screening intervention in rural and urban settings in China. However, important questions remain about how HPV vaccine should be used at population level. For example, what is the optimal age range for vaccination, and whether a catch-up vaccination campaign should accompany the introduction of routine vaccination?

This economic study assumed a large age range from 12 to 55 years to evaluate the impact on the number of cervical cancer and incremental cost-effectiveness ratio (ICER) when adding vaccination to the current screening strategies in China. This wide age range allowed us to identify the age after which vaccination was no longer cost-effective. Since there is wide disparity in cervical cancer incidence and mortality, and unequal availability of health care services between rural and urban areas in China, our assessment will be based on a Markov cohort model adapted to each setting to evaluate lifetime costs and effectiveness of vaccinating girls aged 12 to 55. In such context, we propose to estimate the cost-effectiveness of a 3-dose bivalent HPV vaccination (3DBV) versus current screening practices at ages 12 to 55 years in both rural and urban settings in China.

## Methods

### Model design

The model is a lifetime Markov cohort model (Additional file 1: Figure S1) developed in Microsoft Excel software, based on a previously published model [17]. It consisted of a series of health states in which subjects were located and between which they moved throughout the disease process, reflecting the (simplified) natural history of oncogenic HPV infection to CC.

The Markov model has a cycle time of 1 year and run over life-time according to the mortality rate for women reported by National Bureau of Statistics of China [18]. It consists of three modules: natural history, screening, and vaccination. Overall, it contains 12 different health states for each cycle within which transition occurs each year governed by transition probabilities.

### Data input

This study was approved by the Institutional Review Board (IRB) of Cancer Institute and Hospital of Chinese Academy of Medical Sciences (CICAMS) (Approval No. 13-066/742). The study population was a hypothetical cohort of 12–55 year old girls. The parameters in this modelling study were collected by expert consultation, literature review and data extraction from previous studies conducted by CICAMS in summary forms, so no individual patient information were involved, and informed consent was exempted by CICAMS’ IRB. We incorporated epidemiologic, clinical and economic data in the model to replicate the development of cervical cancer, and interventions such as vaccination, screening and treatment. Major data inputs are showed in Table 1. Some inputs are suited to both rural and urban settings, such as vaccine efficacy, and transition probabilities between states, while others are area-specific, such as HPV infections among women, incidence and mortality of cervical cancer, costs of screening and diagnosis and treatment. We discriminated area-specific data for use in two different scenarios.

**Table 1:** Input data values for base case analysis

Parameter	Base case value		References
	Rural	Urban	
Transition probabilities			
No HPV to HPV	0-0.194	0-0.113	CICAMS pooled data base [30]
HPV to No HPV	0.32-0.61	0.32-0.61	CICAMS pooled data base [30]
HPV to CIN1	0.049	0.049	Moscicki 2001 [25]
CIN1 clearance	0.50	0.50	Van De Velde 2007 [26], Sanders 2003 [27]
CIN1 to CIN2/3	0.121	0.121	Van De Velde 2007 [26], Sanders 2003 [27]
CIN2/3 clearance	0.267	0.267	Melnikow 1998 [28]
CIN2/3 to cancer	0.128	0.128	Melnikow 1998 [28]
CC death rates	0.0699	0.0699	Quinn MA 2006 [29]
Cancer cured	0.212	0.212	Quinn MA 2006 [29]
Screening			
Se of VIA/VILI	0.37-0.55	0.37-0.55	CICAMS pooled data base [30]
Se of Pap smear	0.48-0.52	0.48-0.52	Cuzick J 2006 [36]
Age at 1st screening	35 year	35 year	Assumption
Age at 2nd screening	45 year	45 year	Assumption
Screening coverage	6.25 %	21.5 %	[38–40]
CIN1 treated	34 %	38 %	Delphi panel [22]
CIN1 cured	100 %	100 %	Delphi panel [22]
CIN2/3 treated	83 %	95 %	Delphi panel [22]
CIN2/3 cured	90 %	90 %	Delphi panel [22]
Unit costs(CNY)			
Screening	24 CNY	54 CNY	Delphi panel [22]
CIN1 treatment	367 CNY	681 CNY	Delphi panel [22]
CIN2/3 treatment	2,626 CNY	4,237 CNY	Delphi panel [22]
Cancer treatment	26,715 CNY	26,715 CNY	Delphi panel [22]
Vaccine (3 doses)	1,900 CNY	1,900 CNY	Hong Kong listed price
Vaccine administration (3 doses)	54 CNY	54 CNY	WZ Yu 2006 [23]
Disutility scores^a ^			
CIN1 detected	0.0128	0.0128	[31–35]
CIN 23 detected	0.0128	0.0128	[31–35]
Cancer treated	0.273	0.273	[31–35]
Cancer cured	0.062	0.062	[31–35]
Vaccine efficacy^b^			
Against CC	93.2 %	93.2 %	[3]
Against CIN2/3	64.9 %	64.9 %	[3]
Against CIN1	50.3 %	50.3 %	[3]
General variables			
Discount rate	3 %	3 %	[43]
Age at vaccination (years)	12-55	12-55	Assumption

^a^ Health states No HPV, HPV, CIN 1 undetected and CIN 2/3 undetected have utility = 1 (i.e. no disutility); health states death and death from cervical cancer have utility = 0; ^b^ Irrespective of type

*Se* sensitivity; *CIN* cervical intraepithelial neoplasia; *HPV* human papillomavirus; *CC* cervical cancer; *VIA/VILI* visual inspection with acetic acid/ iodine

### Cost items

A cost study from a societal perspective with a micro-costing approach had been conducted previously by our team to estimate aggregated costs associated with CC [19–21]. We updated that costs to reflect 2013 values and consulted a Delphi panel [22] to confirm/validate/modify the cost estimates. Given the particular situation that most of the patients with diagnostically confirmed CC would seek for treatment in the urban hospitals, only the treatment cost of CC in urban areas were investigated.

The two round Delphi panel, selecting 6 rural and 12 urban clinical gynecologists and epidemiologists from 8 provinces of China, was conducted to assess the costs of screening and treatment, the proportion of patients undergoing the treatment procedure for cervical intraepithelial neoplasia (CIN) and CC. The 8 provinces were chosen from Northern (Beijing, Tianjin, Liaoning), Central (Henan, Shanxi, Jiangsu), Western (Xinjiang) and Southwestern (Chengdu) of China. The two round panels lasted from August 2013 to December 2013 via written questionnaires.

Since HPV vaccine has not been marketed in China, Hong Kong listed price (1900 CNY/3 doses) was used. In addition, we assumed the HPV vaccine could be added into current existing vaccine systems. Introducing a new type of vaccine into national immunization program can make use of existing personnel, equipment, cold chain management, and other management system and don’t need to build new systems. So we just need to consider the increased cost (marginal cost). The cost of HPV vaccine administration was calculated based on the marginal cost of introducing hepatitis B vaccine into China’s expanded programme on immunization, including the employee compensation, surveillance, propaganda, training, supervision, transportation, cold chain and other equipment that were related to the vaccine injection. The incremental vaccine administration cost for an additional dose of hepatitis B vaccine was 17.93 CNY/per child [23]. Finally, we estimated a total of 54 CNY (17.53 × 3 doses ≈ 54) as the administration fee for 3-dose HPV vaccination in the current model. And we found what we estimated from the existing HBV vaccination program (54 CNY) was consistent with the data from Cameroon [24], one of the developing countries, where the administrative cost of HPV vaccine was 8 USD (equivalent to 50 CNY) per 3 doses.

### Transition probabilities and utilities

Data related to the natural history of the disease were derived from literature review [25–29] or calculated from the CICAMS pooled database of 17 population-based studies [30]. The 17 population-based studies included cervical cancer screening studies done in mainland China from 1999 to 2008 with 30,371 women from various parts of China. In this database, every woman had the results of HPV testing recorded, and the screening positive women had the biopsy results included which enabled us to calculate the transition probabilities in natural history. Utilities were obtained from published literature [31–35].

### Screening practice and screening coverage

Currently, Chinese cervical cancer screening program in rural areas sponsored by the government uses VIA/VILI or Pap smear as the primary screening method. In urban areas, most screenings are based on opportunistic exam or through employment-based physical exam using Pap smear. Therefore, we estimated 2/3 of screened women undergoing the Pap smear and the 1/3 undergoing the VIA/VILI in rural areas. In urban areas, we assumed all screened women undergoing Pap smear by the Delphi expert review panel [22]. Sensitivity of VIA/VILI was calculated from the CICAMS pooled database of 17 population-based studies [30], and sensitivity of Pap smear was published data from literature [36]. Both screening scenarios in rural and urban assume twice in a lifetime screening, one at 35 years and one at 45 years according to WHO guidelines on cervical screening in developing countries [37].

A 21.5 % screening coverage in urban areas was assumed as reported in the Human Papillomavirus and Related Cancers, Summary Report Update [38]. From 2012 to 2015, Chinese government is planning to screen about 10 million women in rural areas every year [39]. There were about 1.60 billion women aged 35–64 in rural China according the Sixth National Population Census [40]. So we assumed 6.25 % screening coverage in rural areas in China.

### Vaccine efficacy and vaccine coverage

In the base case analysis, based on the results of the 4-year end of study analysis of the randomised, double-blind PATRICIA trial among HPV naïve girls, a 93.2 %, 64.9 %, 50.3 % overall efficacy against CC, cervical intraepithelial neoplasia 2 or worse (CIN2+), CIN1+ irrespective of types was assumed in the model as a proxy for vaccine effectiveness [3]. For scenario analysis, the effect of a lower VE post-sexual debut was estimated. The lower VE post-sexual debut was assumed as the lower limit of 95 % Confidence Interval (CI) of the reported VE. A cut-off age of 22 years in rural and 21 years in urban was selected to differentiate between pre- and post-exposure based on the median age of sexual debut age in all age groups in China [41].

The vaccination coverage was assumed as 70 % according to the hepatitis B vaccination uptake at three years after the availability of hepatitis B vaccination in China [42].

### Discount rate and study perspective

A 3 % discount rate was used according to the WHO guidelines [43]. We used the same discount rate for health outcomes and cost for analytical purpose.

The model essentially considered the perspective of the health care payer and included only direct medical costs.

### Outcome measures

The main outcome measure used in the model was CC cases and the incremental cost/ quality-adjusted life-years (QALY). According to the recommendation of WHO, a strategy is ‘cost-effective’ when the ICER falls between 1 and 3 times per capita national gross domestic product (GDP) of the country [44]. As there is no GDP for rural and urban areas reported separately in China, the threshold for ICER was assumed to be the 3x national GDP 2013 (125,723 CNY) [45].

### Model validation

The model was validated by comparing the age-specific incidence and mortality of CC in rural and urban China from the model with the ones reported by the National Office for Cancer Prevention and Control [46]. Calibration was performed by adjusting the transition probabilities from persistent CIN2/3 to cervical cancer as well as transition probability to cancer death.

### Base case analysis

The base case analysis was performed on the two modelled cohorts of 12 to 55 years old girls, one cohort assuming to receive an HPV vaccination program with a 70 % coverage and screening, the other cohort assuming to receive only screening. In the regular base case analysis, only girls at one certain age would be vaccinated. For instance, vaccination at age 12 means that only the cohort of 12-year-old girls could be vaccinated; and vaccination at age 54 means that only the cohort of women aged 54 could be vaccinated. The vaccine efficacy was assumed constant across ages in base case analysis. The main outcome considered was CC cases prevented and the incremental cost per QALY gained for the vaccinated cohort compared with the non-vaccination cohort at ages 12 to 55 year in both rural and urban in China.

### Scenario analysis

The HPV-16/18 vaccine had efficacy against infection with HPV-16/18, with higher point estimates in the HPV naïve than in the women with HPV infection [3]. So, the effect of lower vaccine efficacy with post-sexual debut was explored. The lower VE was assumed as the lower limit of 95 % CI of the reported VE.

### Catch up analysis

The other was a catch-up program with additional vaccination cohorts up to age 25. An incremental approach was used to estimate the effect of catch-up scenario, that is, the vaccination of a 12-year-old cohort was compared with a supplementary annual vaccinated cohort in a stepwise manner to the final added vaccination cohort was 25 year old. In other words, regular vaccination would be conducted in the cohort of girls at age 12, and catch-up would be given to the cohorts of females older than age 12. The vaccination coverage rate for each supplementary cohort was fixed at 70 % [42].

## Results

### Model validation

The results showed that the cancer incidence and mortality that produced by the Markov model were consistent with the ones reported by the National Office for Cancer Prevention and Control. Pearson’s correlation analysis was done for incidence and mortality validation. The results showed that the curves of model simulation had no significance with the ones from cancer registry (*r* = 0.985, 0.973, 0.954 and 0.952 for rural incidence, urban incidence, rural mortality and urban mortality respectively) (Additional file 2: Figure S2).

### Base case analysis

**Fig. 1:**
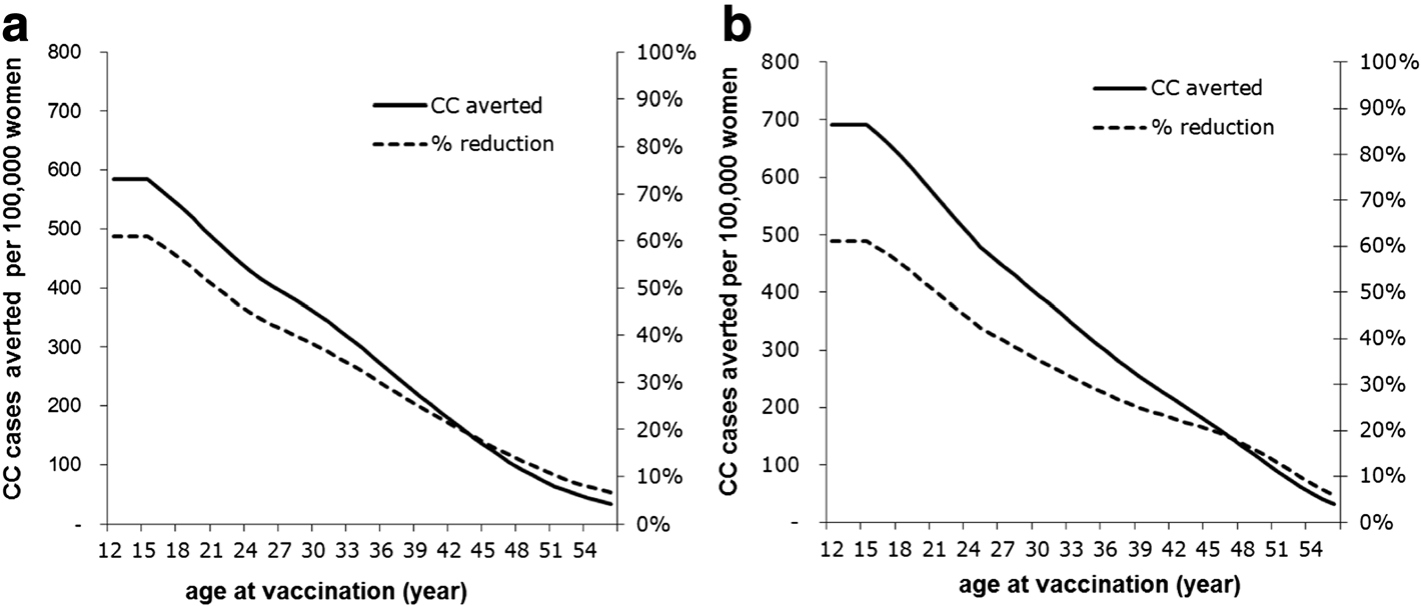
Effect of age at vaccination on cases of CC averted. (**a**: rural; **b**: urban)

The effect of HPV vaccination on CC cases of 100,000 girls and women at ages 12 to 55 was shown in Fig. 1. With 70 % vaccination coverage, a reduction of CC cases over the lifetime of the cohort varying from 585 to 33 in rural and 691 to 32 in urban were estimated at ages 12 to 55, respectively. For example, CC cases prevented in rural and urban was about 61 % when vaccinating at age 12 year old girls (vaccine coverage: 70 %, VE on CC: 93.2 %), while an estimated 22 %, 23 % of CC cases were avoided when vaccinating at age 40. The figure clearly shows that the reduction of CC cases was the largest when vaccinating at early ages but the effectiveness was still substantial at later ages.

**Fig. 2:**
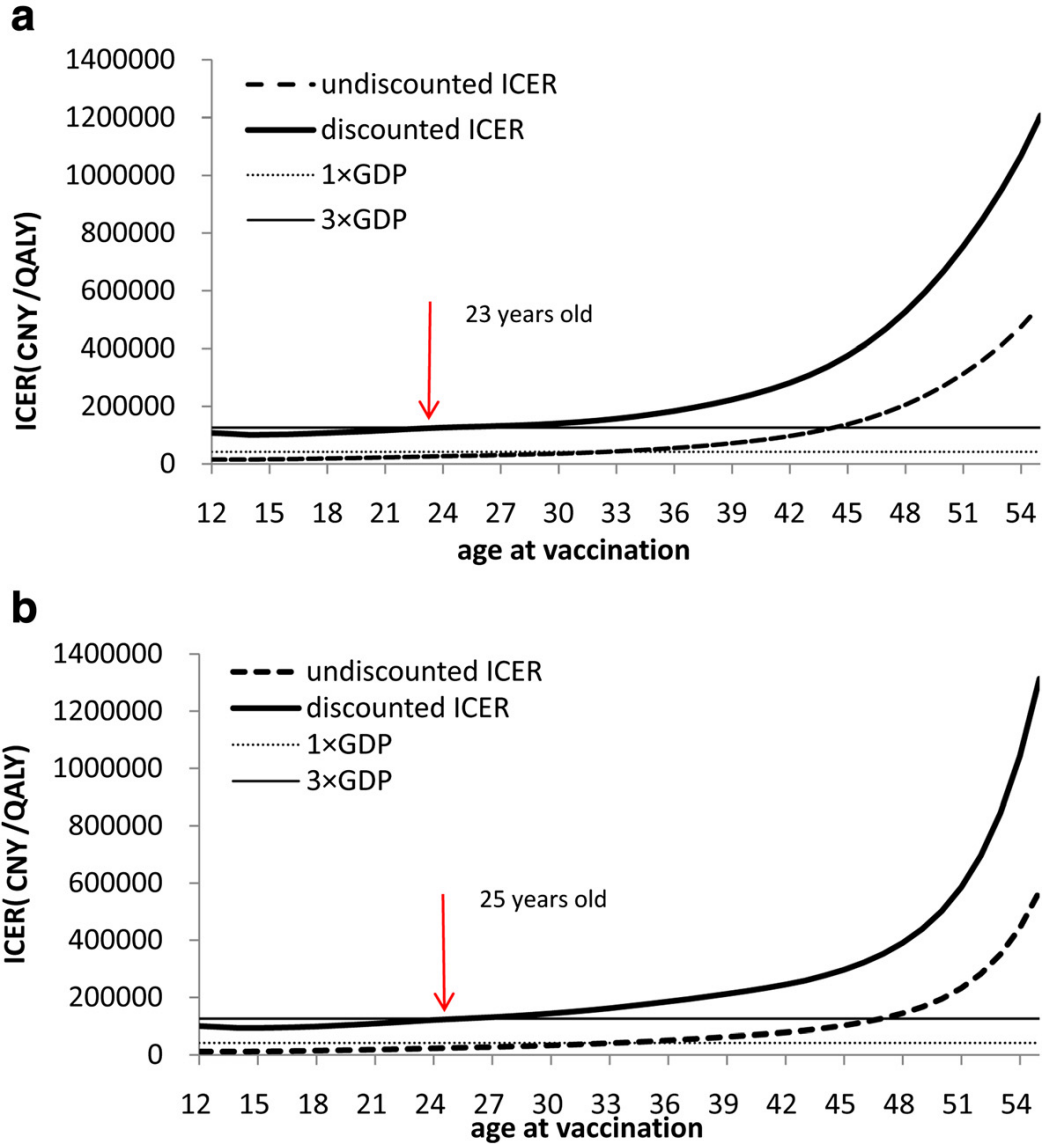
Effect of vaccination age on ICER in rural (**a**) and urban (**b**) China

Figure 2 showed the effect of vaccination age on ICER in rural and urban settings. The results indicated that the estimated ICER of adding vaccination to current screening was lower when vaccinating at younger ages. The undiscounted ICER was the most cost-effectiveness when vaccination occurs at age 12. However, the discounted results show the lowest ICER was found at the age of 14. The discounted ICER results also indicating that HPV vaccination program in girls was cost-effective at any age under 23 in rural and 25 in urban setting under current threshold.

### Scenarios analysis

**Fig. 3:**
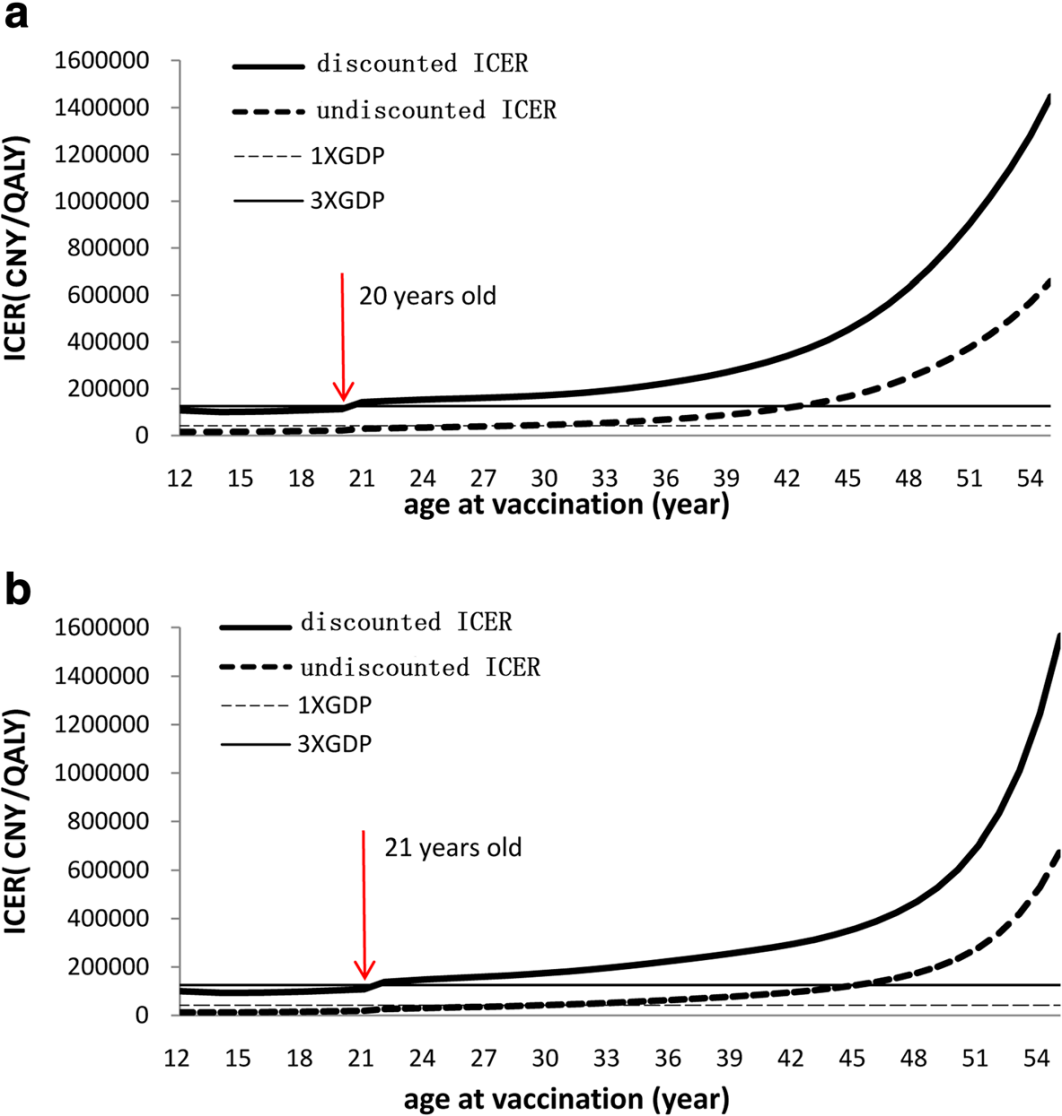
Scenarios analysis - Effect of age at vaccination on ICER in rural (**a**) and urban (**b**)

Scenario analyses with lower VE post-sexual debut confirmed that vaccination remained cost-effectiveness at any age under 20 in rural and 21 in urban, because the ICER was lower than the current threshold, and also indicated that the earlier vaccination was received, the better discounted ICER value was reached at age 14 in rural and urban (Fig. 3).

### Catch up analysis

**Fig. 4:**
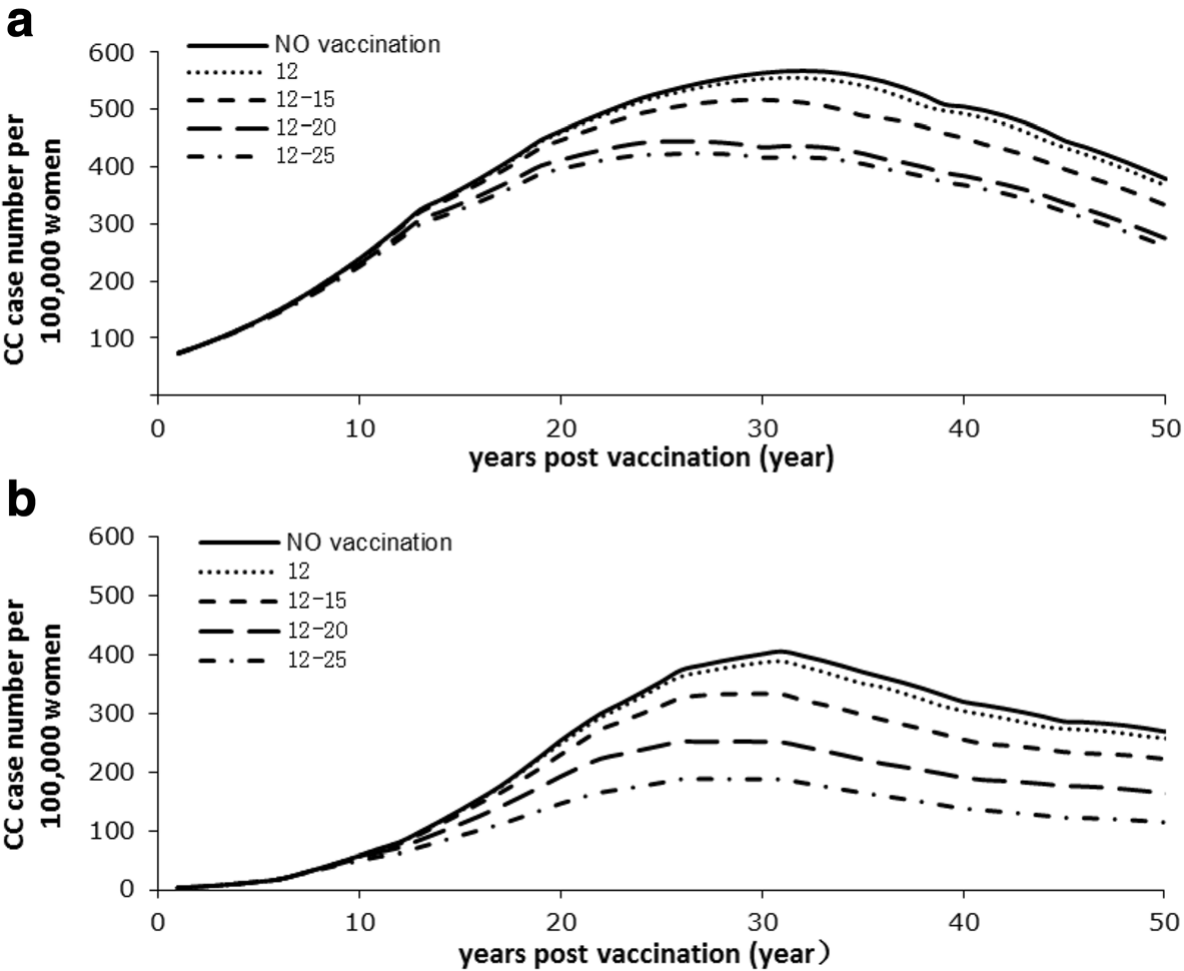
Total CC cases number in vaccinated cohorts 12 to 25 in rural (**a**) and urban (**b**). (12 = vaccinate age 12 only and ages 13–25 not vaccinated; 12–15 = vaccinate ages 12–15 and ages 16–25 not vaccinated; 12–20 = vaccinate ages 12–20 and ages 21–25 not vaccinated; 12–25 = vaccinate ages 12–25)

Figure 4 illustrated the reduction of CC cases due to vaccination in rural and urban. Maximal vaccination reduction of CC cases due to vaccination reached 30 years after vaccination. The additional vaccination cohorts substantially decreased the number of CC cases in both rural and urban settings. The more 'catch-up' cohorts vaccinated, the more CC cases are avoided over the lifetime of the cohorts.

**Fig. 5:**
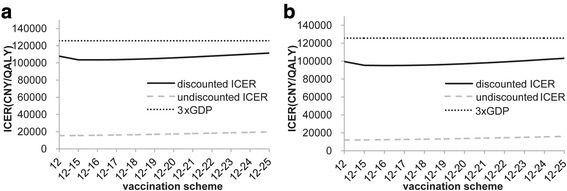
The impact of catch up cohorts on the ICER (**a**: rural; **b**: urban). (12 = vaccinate age 12 only; 12–15 = vaccinate ages 12 and catch-up from 13–15; 12–16 = vaccinate ages 12 and catch-up from 13–16; 12–18 = vaccinate ages 12 and catch-up from 13–18, etc.)

Figure 5 showed the discounted and undiscounted ICER of adding each age vaccination cohort year by year up to age 25. Routine vaccination in 12-year-old girls and a catch-up to age 25 years could still be cost-effective in both rural and urban areas.

## Discussion

To date, epidemiological and economic models to determine the cost-effectiveness of HPV vaccination have been used by government policy-makers for policy deliberations and professional guidelines in many countries. To our knowledge, the present study was the first analysis to assess the effect of vaccination age from 12 to 55 years on cost-effectiveness of HPV vaccination in addition to screening compared with the current screening situation of cervical cancer in China. The evaluation was based on a Markov cohort model adapted to rural and urban settings, simulating the lifetime costs and effects of vaccinating 12 to 55 years old girls and women.

From our study, HPV 16/18 vaccination of younger girls could substantially decrease more CC cases than older women. Vaccination at 12 years old can prevent about 60 % CC cases, whereas vaccination at age 40 can only avert 20 % CC cases. Maximal reduction of CC cases occurred 30 years after vaccination, and the more 'catch-up' cohorts were vaccinated, the more CC cases were avoided in the long-term period. Our findings were consistent with the general consensus that the effectiveness of HPV vaccination decreasing with increasing age of vaccination [10, 31]. Several studies conducted in Europe, North America, South America and Portland indicated that the adolescence and early adulthood had the highest prevalence of HPV infection [47]. 55 % adolescents would be infected by HPV within three years after the sexual debut [25]. Young girls benefit most from a prophylactic vaccine before sexual debut. However, a study included a total of 30,371 women from 17 population-based studies throughout China found that the rates of oncogenic HPV infection are high among women aged 35–39 years in rural and aged 40 or older in urban [48]. Studies in other countries also demonstrated that women over 25 year old still had risks of high HPV incidence [49–51]. Furthermore, immunogenicity data following vaccination with the HPV-16/18 vaccine showed that an age-dependent decrease in antibody titers was observed with increasing age, titers in the younger age group (15–25) remained at least 5-fold higher than those in 46-to-55-year-old women, however, absolute values were high in all age groups, titers in the oldest age group (46–55) remained at least 8-fold higher than those associated with natural infection in 15- to 25-year-old women [52]. In our study, we could conclude that women at all ages will benefit from prophylactic HPV vaccination, but the benefit proportion decreased with age. Rationally, cost-effectiveness must be taken into account when considering HPV vaccination in older women.

The undiscounted data showed cost-effective of HPV vaccination were more favourable at earlier ages of vaccination. Vaccination targeting older girls who may have been infected previously with HPV 16 or 18 may show less benefits compared with young adolescents who have not yet been exposed to HPV [53]. However, the discounted data showed that the most favourable ICER was found at the age of 14 in both rural and urban. That may be because shifting the vaccination to a time closer to the moment of infection will slightly improve the cost-effectiveness results when discounting is applied. Vaccinating at an earlier age involves a longer waiting period before the health effects of the vaccine become apparent compared with vaccinating at an older age [31]. In addition, we found that HPV vaccination program in girls before age 23 in rural and 25 in urban maintained cost-effective at the price of approximately 100 US dollars (1900 CNY per course) for the CC prevention in China. Recently, several clinical trials have shown that not only HPV vaccination of young teenage girls but also vaccination of older girls and women induces high virus-neutralizing antibody titers [52, 54]. We found that catch up program for ages 12–25 years in rural and urban were still cost-effective for a threshold of 3 times national GDP/capita, assuming the vaccine cost per dose was approximately 100 USD. Several countries elected to fund temporary catch-up programs for females up to ages 16 or 18 years (e.g., United Kingdom) and, in fewer countries, up to age 26 years (e.g., Australia) [55]. An alternative approach has been suggested in Italy where concurrent vaccination of 3 cohorts (ages 11, 18 and 25 years) is a more cost-effective strategy in reducing HPV-related cervical diseases among Italian women. However, several studies that have assessed catch-up and routine HPV vaccination programs have found that vaccinating beyond age 22 years is not cost-effective [56–58]. In the U.S., it was proven that HPV vaccination of older women participating in the screening program provides much lower benefits than vaccination of pre-adolescent girls and does not provide good health value for the resources invested, compared with well-accepted health interventions [59]. Explanation for the differences between model analyses have been discussed and are generally attributed to model assumptions, such as the amount of prior exposure to HPV, natural immunity assumptions, transmission dynamics, and vaccine characteristics (e.g., protection among those with prior exposure and cost per dose). A PRIME modelling study [60] assessed differences between countries in terms of cost-effectiveness and health effects, which found that although large between-country disparities exist for HPV vaccination, the effect and cost-effectiveness of vaccinating girls before sexual debut at high coverage can be reasonably predicted from important parameters, such as data for cancer incidence, distribution of HPV type in cancer, and vaccination costs.

The study has limitations. Firstly, the model is a static cohort model, which does not capture the indirect protection resulting from immunization (herd-protection effects). It could not analyse the benefits of herd immunity caused by the reduction of circulation of the infective agent. As a result, the benefits of the vaccination could be underestimated. Secondly, one of the uncertainties we had was whether HPV type replacement took place once vaccination against HPV-16/18 was widespread. The prevalence of HPV-16/18 may fall to very low levels with vaccination. Other oncogenic HPV subtypes currently responsible for relatively few CC case, might fill the niche left by HPV-16/18. To date, most vaccine trials have not seen significant increases in prevalence of non-vaccine types [61], and type replacement is not likely because HPV types were found to occur randomly and to lead to cervical disease independently [62]. Even if type replacement is observed, it may not have important public health implications because HPV 16 and HPV 18 pose much higher cancer risks than other types [63]. The model currently assumed a limited level of replacement by other HPV types. Thirdly, we ignored the effect of natural immunity, because antibodies induced by natural infections are not always protective [51]. Women who have had a previous infection and developed detectable antibody levels may still be at risk of subsequent infections. Lastly, the present model only included cervical cancer and did not take into account vulvar cancer, vaginal cancer, anal and some proportion of oropharyngeal cancer that the HPV vaccine may have efficacy in preventing [64–66]. Should these diseases be included in the evaluation, the protection offered by HPV vaccination would be wider, and could lead to a lower ICER than the present analysis. These results are thus likely to provide conservative estimates.

## Conclusion

We conclude that 14 years old maybe the most favourable vaccination age in rural and urban, and a bivalent HPV vaccination program in girls before age 23 in rural and 25 in urban setting was shown to be cost-effective strategies for the prevention of CC in China. Catch-up programmes that extend to age 25 years in rural and urban could still be cost-effectiveness.

## Additional files

Additional file 1: Figure S1. Lifetime cohort Markov model adapted to the rural and urban settings in China. (Note: CIN, cervical intraepithelial neoplasia; CIN1onc, cervical intraepithelial neoplasia 1 oncogenic; HPV, human papillomavirus; HPVonc, oncogenic HPV infection; NoHPVonc, no oncogenic HPV infection). (JPG 24 kb) Additional file 2: Figure S2. Comparison of GCM with the Chinese Cancer Registry Annual Report 2013. (A: CC incidence in rural, B: CC incidence in urban, C: CC mortality in rural, D: CC mortality in urban). (TIF 8248 kb)

## Abbreviations

3DBV: 3 doses of bivalent HPV vaccination
CC: cervical cancer
CI: confidence interval
CICAMS: Cancer Institute, Chinese Academy of Medical Sciences
CIN: Cervical Intraepithelial Neoplasia
CNY: China Yuan
GDP: Gross Domestic Product
HPV: human papillomavirus
ICER: Incremental Cost-effectiveness ratio
PATRICIA: PApilloma TRIal against Cancer In young Adults
QALYs: Quality-adjusted Life-years
USD: United States Dollar
VE: vaccine efficacy
VIA/VILI: Visual Inspection of Acetic Acid/Lugol’s Iodine
WHO: World Health Organization
