# Supplementary figures and images for: Effect of vaccination age on cost-effectiveness of human papillomavirus vaccination against cervical cancer in China
Source: BMC Cancer. 2016 Feb 26;16:164. doi: 10.1186/s12885-016-2207-3 (PMC4768405; doi:10.1186/s12885-016-2207-3)

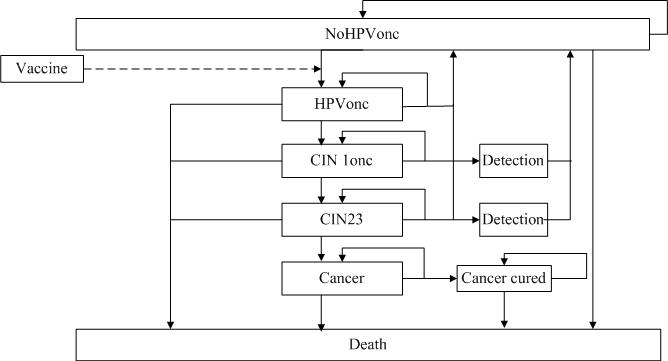

Supplement: Additional file 1: Figure S1. — Lifetime cohort Markov model adapted to the rural and urban settings in China. (Note: CIN, cervical intraepithelial neoplasia; CIN1onc, cervical intraepithelial neoplasia 1 oncogenic; HPV, human papillomavirus; HPVonc, oncogenic HPV infection; NoHPVonc, no oncogenic HPV infection). (JPG 24 kb) [file 12885_2016_2207_MOESM1_ESM.jpg]

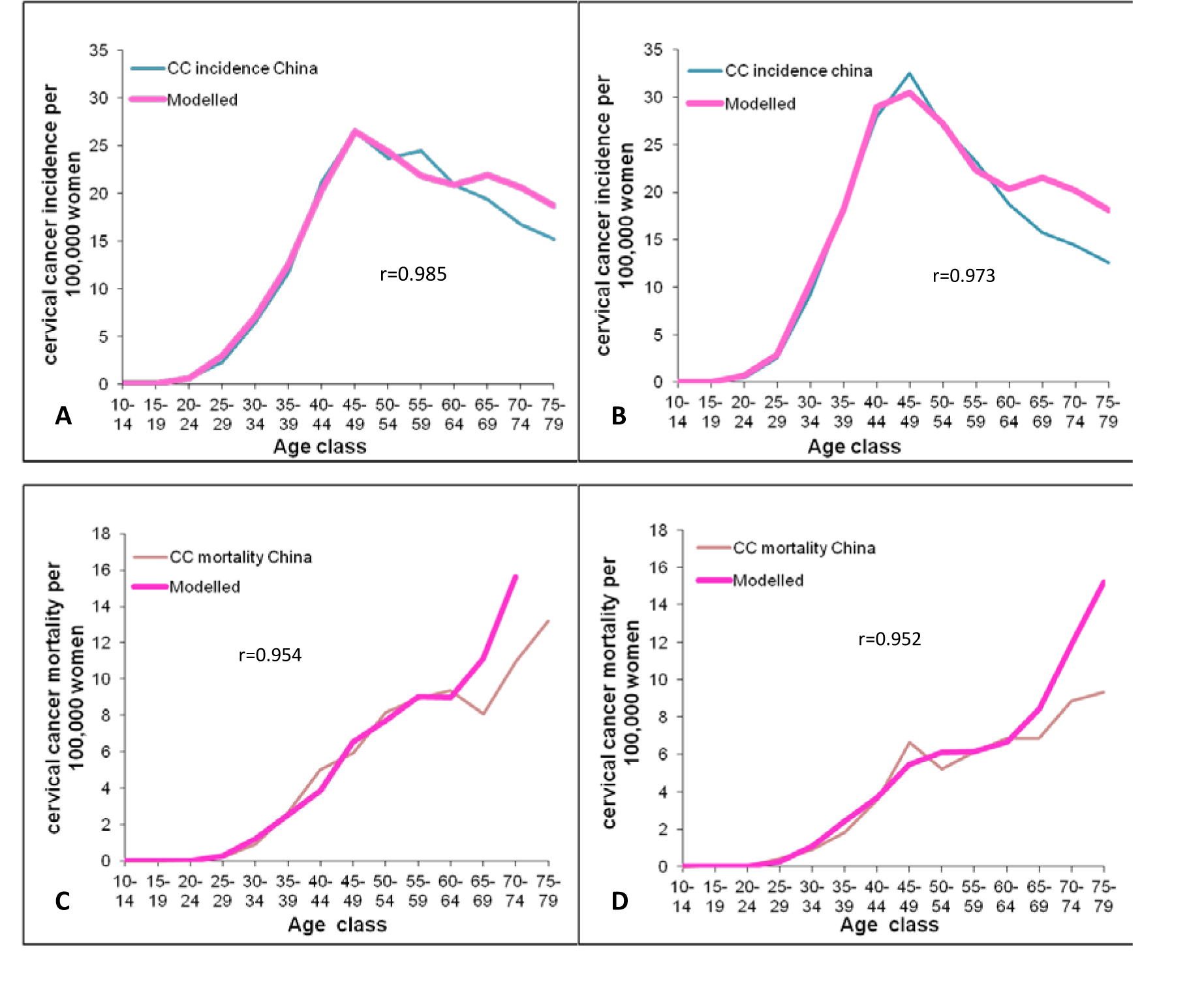

Supplement: Additional file 2: Figure S2. — Comparison of GCM with the Chinese Cancer Registry Annual Report 2013. (A: CC incidence in rural, B: CC incidence in urban, C: CC mortality in rural, D: CC mortality in urban). (TIF 8248 kb) [file 12885_2016_2207_MOESM2_ESM.tif]
